# Traceability and dispersion of highly toxic soluble phases from historical mine tailings: insights from Pb isotope systematics

**DOI:** 10.1007/s10653-024-02180-3

**Published:** 2024-08-24

**Authors:** Rafael Del Rio-Salas, Verónica Moreno-Rodríguez, René Loredo-Portales, Sergio Adrián Salgado-Souto, Martín Valencia-Moreno, Lucas Ochoa-Landín, Diana Romo-Morales

**Affiliations:** 1 Estación Regional del Noroeste, Instituto de Geología, Universidad Nacional Autónoma de México, Colosio y Madrid s/n, 83000 Hermosillo, Sonora México; 2 Laboratorio Nacional de Geoquímica y Mineralogía-LANGEM, 04510 Ciudad de México, México; 3 Ingeniería en Geociencias, Universidad Estatal de Sonora, Av. Ley Federal del Trabajo s/n, Col. Apolo, 83100 Hermosillo, Sonora México; 4 Escuela Superior de Ciencias de la Tierra, Universidad Autónoma de Guerrero, Ex-hacienda de San Juan Bautista s/n, 40323 Taxco el Viejo, Guerrero México; 5 División de Ciencias Exactas y Naturales, Departamento de Geología, Universidad de Sonora, Rosales y Encinas s/n, 83000 Hermosillo, Sonora México

**Keywords:** Historical mine tailings, Pb isotopes, PTE dispersion, Traceability, Efflorescence salts

## Abstract

Dispersion of potentially toxic elements associated with efflorescent crusts and mine tailings materials from historical mine sites threaten the environment and human health. Limited research has been done on traceability from historical mining sites in arid and semi-arid regions. Pb isotope systematics was applied to decipher the importance of identifying the mixing of lead sources involved in forming efflorescent salts and the repercussions on traceability. This research assessed mine waste (sulfide-rich and oxide-rich tailings material and efflorescent salts) and street dust from surrounding settlements at a historical mining site in northwestern Mexico, focusing on Pb isotope composition. The isotope data of tailings materials defined a trending line (R^2^ = 0.9); the sulfide-rich tailings materials and respective efflorescent salts yielded less radiogenic Pb composition, whereas the oxide-rich tailings and respective efflorescent salts yielded relatively more radiogenic compositions, similar to the geogenic component. The isotope composition of street dust suggests the dispersion of tailings materials into the surroundings. This investigation found that the variability of Pb isotope composition in tailings materials because of the geochemical heterogeneity, ranging from less radiogenic to more radiogenic, can add complexity during environmental assessments because the composition of oxidized materials and efflorescent salts can mask the geogenic component, potentially underestimating the influence on the environmental media.

**Supplementary Information:**

The online version contains supplementary material available at 10.1007/s10653-024-02180-3.

## Introduction

Efflorescence salts are naturally occurring secondary minerals (e.g., gypsum, rozenite, coquimbite, szomolnokite) in various environments, including mine waste. In the context of mine waste, the efflorescent salts can accumulate and momentarily immobilize metals, which can be released later in solution, as they commonly consist of highly soluble phases (Lottermoser, 2017). Additionally, efflorescent salts are characterized by low cohesion and density, facilitating decrepitation, lifting, and wind dispersion (Del Rio-Salas et al., 2019; Punia, 2021). Therefore, although ephemeral, they are quite effective in dispersing potential toxic elements (PTE) into the environment.

Among the different types of mine waste (mining, mineral processing, metallurgical, etc.), mine tailings impoundments are considered one of the largest and most dangerous industrial infrastructures worldwide (Davies, 2002). Mine tailings are constructed to confine the waste during the mine’s operational life and after closure. They contain fine-grained materials, including minerals, rock fragments, sediments, chemical reactants, and water. Tailings impoundments generated during historical mining commonly contain higher PTE contents than those from active mines due to less efficient metallurgical processes and laxer environmental standards at the time, among other reasons (e.g., Del Rio-Salas et al., 2019). Particularly in developing countries, historical tailings impoundments lack containment, monitoring, and remediation programs and have become part of the landscape (e.g., Peña-Ortega et al., 2019).

PTE content in historical mine tailings is related to metal commodities allocated within sulfide minerals after processing. Exposure to air and humidity triggers the breakdown of sulfides through oxidation processes, producing acidic waters enriched in sulfate and PTE, a phenomenon widely known as acid mine drainage (AMD) (Lottermoser, 2010, 2017). AMD is critical in arid and semi-arid environments since high temperatures, torrential rains, high evaporation rates, and capillarity action favor the migration of PTE-bearing fluids to the tailings surface, precipitating the efflorescent salts (Khorasanipour, 2015) composed mainly by sulfates, in addition to chlorides, nitrates, among other mineral species.

Understanding the dispersion of tailings materials and efflorescent salts is essential not only because of their high capacity to contaminate different environmental media such as soil, water, and dust but also because of their high potential toxicity to humans (Gonzalez et al., 2014; Loredo-Jasso et al., 2021; Martínez-López et al., 2021; Pérez-Sirvent et al., 2016; Punia, 2021). Specifically, efflorescent salts and relatively high PTE contents related to mine tailings have been identified in sediments (García-Lorenzo et al., 2012) and dust from rural settlements near historical mining sites (Del Rio-Salas et al., 2019). In the latter scenario, rural dust can serve as the final fate for PTE associated with mine tailings and efflorescent salts; detecting efflorescent salts and high PTE concentration in rural dust underscores their dispersal capacity despite their temporary nature.

Among different techniques to assess dispersion, Pb isotope systematics has effectively traced Pb in various research fields, including petrogenesis, tectonics, ore deposits, anthropology, forensic sciences, environmental sciences, etc. Lead has four naturally occurring stable isotopes (^204^Pb, ^206^Pb, ^207^Pb, and ^208^Pb); ^204^Pb is non-radiogenic, while ^206^Pb, ^207^Pb, and ^208^Pb are the decay products of ^238^U, ^235^U, and ^232^Th, respectively (Dickin, 1995). Lead does not fractionate, and particularly in environmental studies, processes such as emission, transport, and deposition do not affect the isotope composition, enabling source identification (geogenic versus anthropogenic) and tracing dispersion of PTE in environmental media (e.g., dust, particulate matter, soils, sediments, plants, mine waste) and organisms (i.e., Dong et al., 2020; Fry et al., 2020; Lee et al., 2019; McPartland et al., 2020; Mihaljevič et al., 2019; Nazarpour et al., 2019; Pelletier et al., 2020; Romo-Morales et al., 2020; Seleznev et al., 2022; Zhao et al., 2019).

Despite the proven effectiveness of Pb isotope systematics in studying PTE dispersion from mining sites, more research is needed to improve the traceability of PTE related to efflorescence crusts from historical mining sites in arid and semi-arid environments. The present study uses the Pb isotope composition to investigate the traceability of mine tailings and their respective efflorescent salts. A historical mine tailings deposit located in the semi-arid region of northwestern Mexico was selected to address this issue, considering the reported efflorescence salts and high PTE concentrations (Del Rio-Salas et al., 2019). The objectives of this investigation were: (i) to determine the Pb isotope composition of historical mine tailings and rural dust from surrounding settlements to identify the dispersion of PTE related to the mine tailings deposit, and (ii) to determine the Pb isotope composition of the oxide-rich and sulfide-rich tailings materials, as well as their respective efflorescent crusts, to identify similarities with the geogenic end-member and their implications. The findings provide information regarding the dispersion of efflorescent salts and mine tailings materials, the challenges regarding sourcing (geogenic vs anthropogenic), and the traceability of PTE from historical mine tailings into the surrounding environment.

## Materials and methods

### Study site

**Fig. 1:**
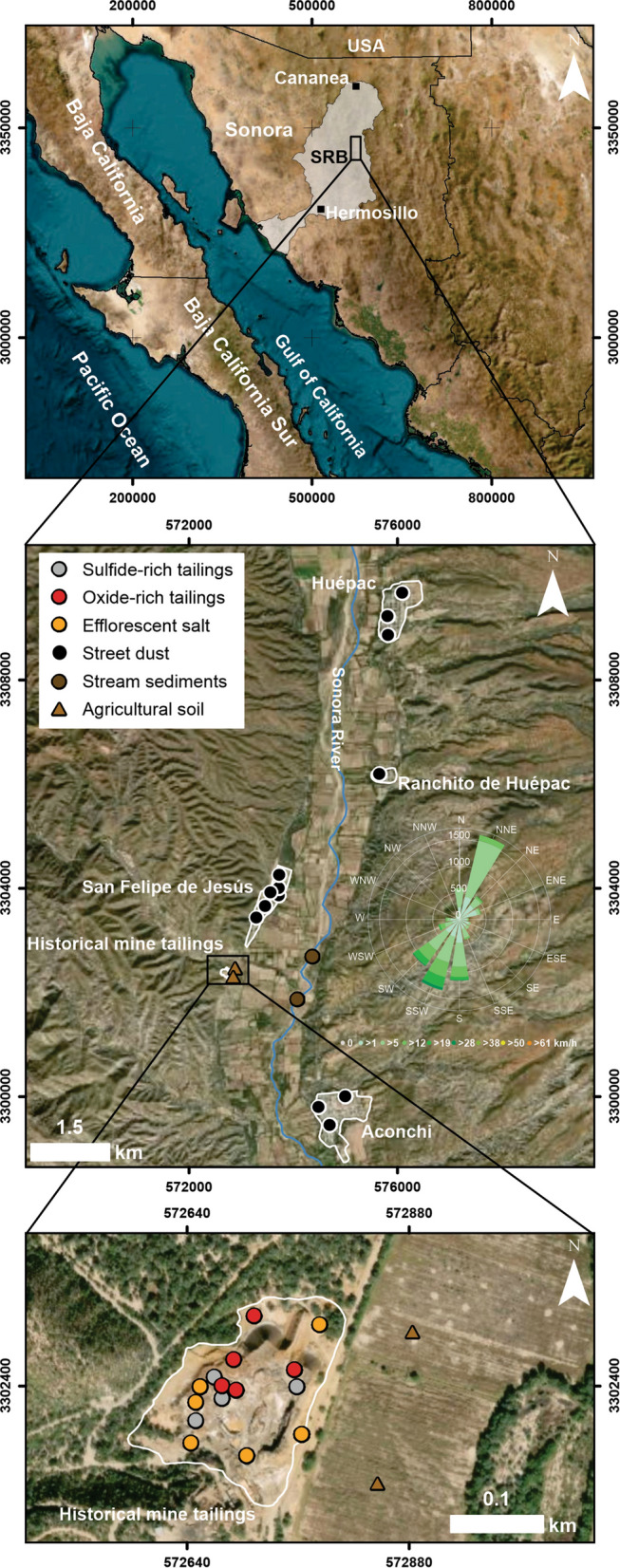
Map depicting the location and sampling points of San Felipe de Jesús area and nearby towns in central Sonora, Mexico. SRB: Sonora River Basin

**Fig. 2:**
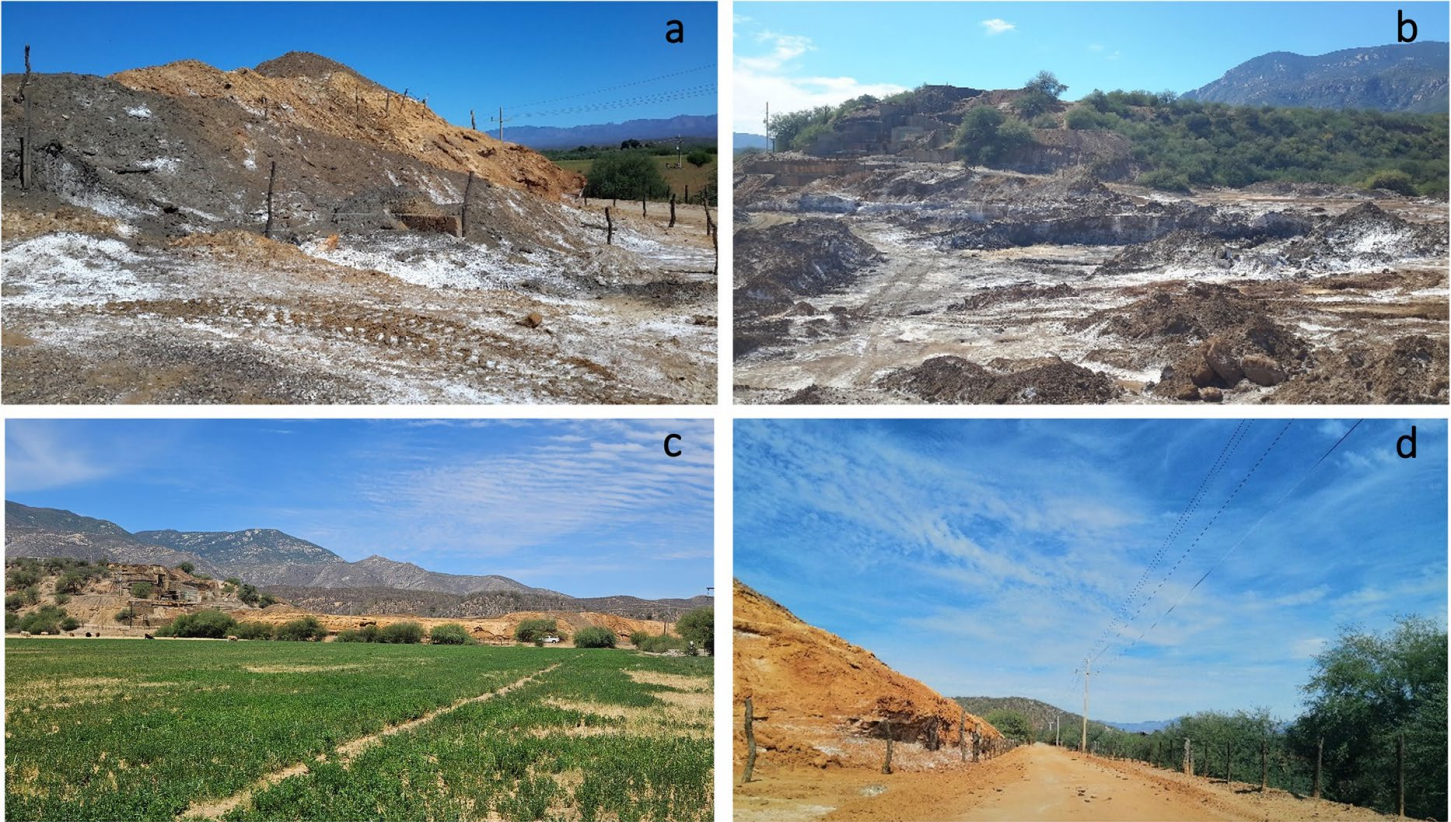
Pictures depicting some features of the historical mine tailings near San Felipe de Jesús settlement. **a** Sulfide-rich (lower part) and oxide-rich (upper part) tailings; note the development of efflorescent crusts (white crusts) over the sulfide-rich materials. **b** View of the tailings deposit with efflorescent crusts developed over the surface. **c** Active agricultural soils next to the tailings deposit. **d** Road next to tailings deposit that connects to the San Felipe de Jesús town

San Felipe de Jesús town is located in the Sonora River Basin (northwestern Mexico). Mining, agriculture, and cattle raising represent the most relevant activities in this region. San Felipe de Jesús, a small settlement with ~ 400 inhabitants (INEGI, 2010), is neighboring Huépac, Ranchito de Huépac, and Aconchi towns in north-central Sonora; predominant wind directions are north-northeast and south-southwest (Fig. 1). A historical small-scale metallurgical facility and a small (~ 140 × ~ 160 m) mine tailings deposit are located approximately 500 m south of San Felipe de Jesús. The deposit contains ~ 209 tons of waste accumulated since 1920 after the exploitation of skarn mineralization (Ag, Pb, Cu, and Zn) from several small underground mines in the region (Espinoza, 2012); minerals hosting the mineralization included galena (PbS), sphalerite (ZnS), arsenopyrite (FeAsS), pyrrhotite (Fe_7_S_8_), chalcopyrite (CuFeS_2_), among other sulfides. The tailings material is fine-grained, unconsolidated, and lacks vegetation, which potentially favors hydric and wind dispersion of material with relatively high concentrations of As (6213–10,098 μg/g), Cu (338–491 μg/g), Mn (16,255–29,519 μg/g), Pb (10,464–14,161 μg/g), and Zn (8285–60,709 μg/g) to surroundings (Del Rio-Salas et al., 2019). The deposit is reddish in the more external parts because of the relative abundance of oxide minerals and grayish in the internal zones because of the relative abundance of sulfide minerals. Development of efflorescent crusts over both types of phases (oxide- and sulfide-rich) materials was observed (Fig. 2) and are also characterized by having high concentrations (As: 1305–16,756 μg/g; Cu: 1052–5,691 μg/g; Mn: 41,562–117,418 μg/g; Pb: 831–8672 μg/g; and Zn: 163,909–176,218 μg/g) (Del Rio-Salas et al., 2019). The most abundant sulfate minerals identified were gypsum, jarosite, kieserite, epsomite, szomolnokite, rozenite, coquimbite, copiapite, starkeyite, beudantite, kieserite, anglesite, among others (Del Rio-Salas et al., 2019). More studies have also targeted the study area to determine PTE mobility to the surrounding media (Archundia et al., 2021; Loredo-Portales et al., 2020), the speciation and oxidation state of Mn (Morales-Pérez et al., 2021), and the distribution of heavy metals in surrounding agricultural soils (González‑Méndez et al., 2022).

Among the several mining developments along the Sonora River basin, the most outstanding mining zone is located at the northernmost part of the Sonora River basin, represented by the Buenavista del Cobre mine (formerly known as the Cananea mine), the largest porphyry copper mine in Mexico. This mine spilled ~ 40,000 m^3^ of Fe–Cu acid solution along the river in 2014 (Calmus et al., 2018). After that, several studies assessed the impact (e.g., Díaz-Caravantes et al., 2016; Escobar-Quiroz et al., 2019; Romero-Lázaro et al., 2019; Romo-Morales et al., 2020).

### Sample collection and preparation

A total of 42 samples were collected for the investigation. Samples from oxide-rich tailings (ORT; n = 8), sulfide-rich tailings (SRT; n = 7), and efflorescent crust collected on both tailings materials (n = 9) were collected from the historical mine tailings near the San Felipe de Jesús town. Moreover, street dust samples were collected from the San Felipe de Jesús (n = 8), Huépac (n = 3), Ranchito de Huépac (n = 1), and Aconchi (n = 3) settlements. Also, agricultural soils (n = 2) from surrounding fields were collected. Additionally, a pyrrhotite sample was collected from the closed El Gachi mine, located ~ 50 km north of the area; this sample was considered in this study since the material that was exploited from this mine was sent to the metallurgical facility of San Felipe de Jesús; therefore, this sample may be representative of the material treated in such facility. A mineralized porphyry rock sample and pyrite sample related to the Cu mineralization from the Buenavista del Cobre mine were collected to compare the Pb isotope signature with the samples from the study area.

The oxide- and sulfide-rich mine tailings were collected using a stainless shovel and stored in high-density plastic bags with an airtight seal. Efflorescent crusts were collected using stainless steel tweezers and stored in plastic bottles. Street dust was collected using a broom and dustpan, and samples were stored in high-density plastic bags. The samples were air-dried (if needed) and later were sieved to obtain the fraction < 20 µm; the sieves were ultrasonic cleaned and dried between each sample preparation. The fraction < 20 µm of each sample was powdered using an agate mortar; the mortar was cleaned with powdered quartz and MQ-water before each sample preparation. The sulfide samples were carefully picked and ground using an agate mortar; the mineralized porphyry rock sample was crushed and powdered in a Retsch S100 centrifugal agate ball mill.

### Lead isotope ratios

The acids used during the digestion and treatment of samples for measuring Pb isotope ratios were distilled twice, and solutions were prepared with ultrapure Milli-Q water. About 0.5 g of sample (e.g., mine tailings, efflorescent crusts, street dust, sulfide) was digested with aqua regia overnight in Savillex Teflon containers. The rock sample was digested using a mixture of hydrogen fluoride, nitric acid, hydrochloric acid, and perchloric acid. After digestion, the samples were evaporated and reconstituted with 8 M HNO_3_ for a chromatography procedure using Sr-Spec™ resin. Details on sample treatment and measurements are detailed in Thibodeau et al. (2007) and Thibodeau et al. (2012). The Pb isotope ratios were measured in an Inductively Coupled Plasma Mass Spectrometry Multi-collector (MC-ICP-MS) from GV Instruments at the University of Arizona. During the measurements, a total of 185 replicates of reference material were performed. The certified reference material used was NIST (NBS) 981. Accuracy and precision of all isotope ratios ranged from 99.97 to 100.03 and from 4.28E-05 to 4.72E-03, respectively. Errors during the measurements ranged ^206^Pb/^204^Pb = 16.9405 (± 0.0034–0.0036 2σ), ^207^Pb/^204^Pb = 15.4963 (± 0.0033–0.0038 2σ), and ^208^Pb/^204^Pb = 36.7219 (± 0.0089–0.0099 2σ).

## Results and discussion

### Pb isotope composition

The source and dispersion of PTE were assessed using the Pb isotope systematics. Table 1 shows the Pb isotope compositions of the oxide-rich, sulfide-rich, and respective efflorescent crust materials of the historical mine tailings deposit near San Felipe de Jesús town, in addition to the Pb isotope data of street dust collected from surrounding settlements (Fig. 1). Table 1 also includes the Pb isotope data from mineralization sample collected in the inactive El Gachi mine, and the available Pb isotope composition of lithological units outcropping in the region reported by González-León et al. (2011) and González-Becuar et al. (2017), which are representative of the geogenic component of the area.

**Table 1:** Lead isotope compositions of historical mine tailings, efflorescent crusts, and street dust of San Felipe de Jesús area, and mineralized and other environmental matrices of the region, in northwestern Mexico

Sample	Comment	206Pb/207Pb	208Pb/207Pb	206Pb/204Pb	207Pb/204Pb	208Pb/204Pb	Reference
JNO3	Sulfide-rich tailings	1.209	2.474	18.922	15.649	38.713	Present study
JNO4	Sulfide-rich tailings	1.207	2.474	18.885	15.647	38.715	Present study
JNO15	Sulfide-rich tailings	1.207	2.474	18.891	15.646	38.707	Present study
JNO8	Sulfide-rich tailings	1.209	2.474	18.912	15.648	38.715	Present study
JG16-02	Sulfide-rich tailings	1.206	2.474	18.859	15.639	38.693	Present study
JSF15-08	Sulfide-rich tailings	1.220	2.473	19.113	15.672	38.761	Present study
JNO10	Sulfide-rich tailings	1.206	2.474	18.860	15.644	38.709	Present study
JSF1509	Oxide-rich tailings	1.228	2.472	19.260	15.683	38.771	Present study
HO5	Oxide-rich tailings	1.229	2.472	19.287	15.689	38.781	Present study
JO1	Oxide-rich tailings	1.227	2.473	19.252	15.685	38.781	Present study
JO2	Oxide-rich tailings	1.207	2.474	18.882	15.644	38.703	Present study
JO4	Oxide-rich tailings	1.221	2.474	19.135	15.671	38.768	Present study
JR16-01	Oxide-rich tailings	1.207	2.474	18.883	15.642	38.699	Present study
JR16-02	Oxide-rich tailings	1.211	2.474	18.943	15.649	38.714	Present study
JM16-03	Oxide-rich tailings	1.210	2.474	18.940	15.651	38.719	Present study
JSF15-03	Efflorescent crusts on SRT	1.211	2.474	18.952	15.650	38.714	Present study
JSF15-10	Efflorescent crusts on SRT	1.211	2.474	18.943	15.649	38.712	Present study
EF16-01	Efflorescent crusts on SRT	1.209	2.474	18.918	15.644	38.704	Present study
JSF15-14	Efflorescent crusts on ORT	1.222	2.473	19.149	15.671	38.754	Present study
ENO1	Efflorescent crusts on ORT	1.210	2.474	18.938	15.651	38.717	Present study
EO2	Efflorescent crusts on ORT	1.228	2.472	19.260	15.684	38.772	Present study
EO1	Efflorescent crusts on ORT	1.229	2.472	19.287	15.690	38.792	Present study
JSF15-10	Efflorescent crusts on ORT	1.209	2.474	18.925	15.654	38.730	Present study
JSF15-12B	Efflorescent crusts on ORT	1.229	2.472	19.276	15.688	38.788	Present study
ACN-01	Street dust (Aconchi)	1.208	2.466	18.915	15.661	38.614	Present study
ACN-02	Street dust (Aconchi)	1.215	2.472	19.031	15.661	38.709	Present study
ACN-03	Street dust (Aconchi)	1.209	2.468	18.939	15.661	38.648	Present study
HPC-01	Street dust (Huepac)	1.220	2.472	19.125	15.676	38.745	Present study
HPC-02	Street dust (Huepac)	1.208	2.474	18.910	15.649	38.710	Present study
HPC-02	Street dust (Huepac)	1.209	2.473	18.905	15.643	38.693	Present study
HPC-03	Street dust (Huepac)	1.218	2.469	19.083	15.667	38.681	Present study
RHPC-01	Street dust (Ranchito de Huepac)	1.222	2.474	19.156	15.673	38.778	Present study
PSF16-01	Street dust (San Felipe de Jesus)	1.217	2.472	19.064	15.666	38.731	Present study
PSF16-02	Street dust (San Felipe de Jesus)	1.220	2.473	19.121	15.669	38.744	Present study
PSF16-03	Street dust (San Felipe de Jesus)	1.217	2.473	19.056	15.661	38.727	Present study
PSF16-04	Street dust (San Felipe de Jesus)	1.210	2.474	18.925	15.648	38.708	Present study
PSF16-05	Street dust (San Felipe de Jesus)	1.230	2.472	19.290	15.685	38.779	Present study
PSF16-06	Street dust (San Felipe de Jesus)	1.228	2.472	19.250	15.684	38.773	Present study
PSF16-07	Street dust (San Felipe de Jesus)	1.226	2.473	19.230	15.682	38.774	Present study
PSF16-08	Street dust (San Felipe de Jesus)	1.220	2.473	19.104	15.665	38.740	Present study
SSF15-15	Agricultural soil	1.227	2.473	19.244	15.682	38.784	Present study
SSF15-18	Agricultural soil	1.229	2.473	19.270	15.684	38.795	Present study
GA-12a	Pyrrhotite from El Gachi mine	1.205	2.475	18.859	15.645	38.720	Present study
7–25-09–3	El Oquimonis granite (42 Ma)	1.228	2.480	19.264	15.684	38.898	Gonzalez-Becuar et al. (2017)
EGB12-33	El Palofierral orthogneiss (> 72 Ma	1.238	2.470	19.408	15.680	38.731	Gonzalez-Becuar et al. (2017)
EGB12-23	El Gato diorite (72 Ma)	1.228	2.472	19.226	15.656	38.699	Gonzalez-Becuar et al. (2017)
9–27-09–2	Las Mayitas granodiorite (20 Ma)	1.220	2.477	19.105	15.656	38.781	Gonzalez-Becuar et al. (2017)
11–21-09–3	El Garambullo gabbro (20 Ma)	1.219	2.476	19.091	15.666	38.783	Gonzalez-Becuar et al. (2017)
9–30-09–4	La Aurora Tonalita	1.225	2.481	19.185	15.667	38.867	Gonzalez-Leon et al. (2011)
9–29-09–3	Granito Huepac	1.236	2.480	19.400	15.696	38.923	Gonzalez-Leon et al. (2011)
BCS1	BCM spill (2014)	1.185	2.462	18.657	15.746	38.765	Romo-Morales et al. (2020)

SRT, sulfide-rich tailings; ORT, oxide-rich tailings; BCM, Buenavista del Cobre mine; Ma, million years

**Fig. 3.**
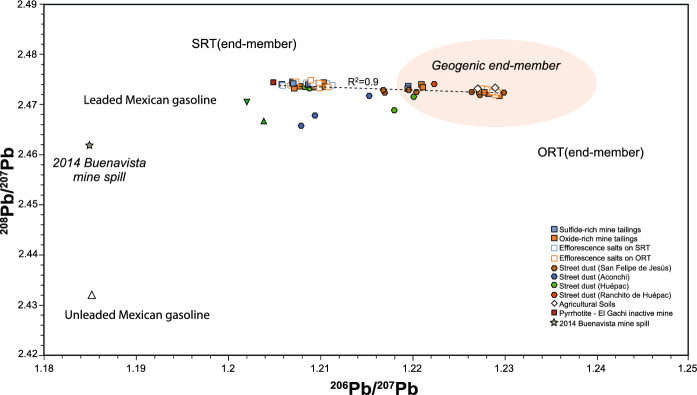
^206^Pb/^207^Pb versus ^208^Pb/^207^Pb diagram showing the composition of historical mine tailings from San Felipe de Jesús and street dust from nearby settlements. The geogenic end-member field is shown according to the representative lithology of the study area (González-Becuar et al., 2017; González-León et al., 2011). Also shown is the isotope data of the 2014 Buenavista del Cobre mine spill (Romo-Morales et al., 2020) and unleaded (Morton-Bermea et al., 2011) and leaded (Sañudo-Wilhelmy & Flegal, 1994) Mexican gasoline

A clear tendency line is formed (R^2^ = 0.9) by the isotope compositions of the mine tailings samples (Fig. 3), where the sulfide-rich materials represent the less radiogenic Pb component (^206^Pb/^207^Pb ≈ 1.206 and ^208^Pb/^207^Pb ≈ 2.474). In contrast, the more radiogenic member is represented by efflorescence salts and oxide-rich materials (^206^Pb/^207^Pb ≈ 1.229 and ^208^Pb/^207^Pb ≈ 2.472). Along this tendency is the isotope composition of efflorescent salts formed over both types of tailings (sulfide- and oxide-rich). The less radiogenic ratios of the sulfide-rich tailings are similar to the Pb isotope composition of a pyrrhotite sample from the inactive El Gachi mine, whose material was processed in the metallurgical facility of the study area. The available Pb isotope data of the lithological units outcropping south and north of the research site are plotted as a reference, whose compositions are the most radiogenic (^206^Pb/^207^Pb = 1.219–1.238 and ^208^Pb/^207^Pb = 2.470–2.481; Fig. 3) and represent the geochemical background (geogenic end-member) since these rocks are widespread in the region (Calmus et al., 2018; González-Becuar et al., 2017; González-León et al., 2011). The composition of dust collected from surrounding settlements is closely related to the tendency line formed by the mine tailings and efflorescent salts (Fig. 3), particularly the dust from San Felipe de Jesús, the nearest settlement to the historical mine tailings. The similarity in the isotope composition may suggest the influence of the mine waste.

Moreover, to provide contextualization from the perspective of environmental incidents in the region, the isotopic composition of Pb from the 2014 spill at Buenavista del Cobre mine (Romo-Morales et al., 2020) is included in Fig. 3; the composition of the spill is less radiogenic than the tendency line defined by the mine tailings, rural dust, and the geogenic component field. Also, Fig. 3 includes the Pb isotope composition of leaded Mexican gasoline (Sañudo-Wilhelmy & Flegal, 1994), whose composition is slightly less radiogenic than that of sulfide-rich materials end-member. Moreover, unleaded Mexican gasoline is characterized by a less radiogenic nature (Morton-Bermea et al., 2011) (Fig. 3). The Pb isotope compositions of Mexican gasoline do not explain the compositions determined in rural dust of studied settlements.

**Fig. 4.**
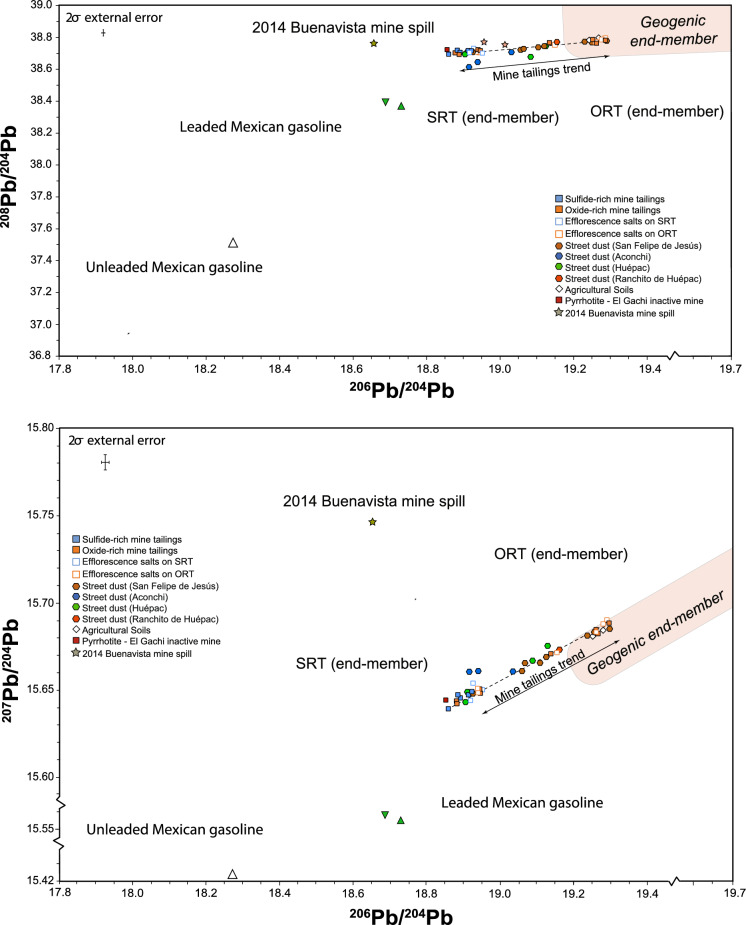
^206^Pb/^204^Pb versus ^207^Pb/^204^Pb and ^206^Pb/^204^Pb versus ^208^Pb/^204^Pb diagrams of historical mine tailings from San Felipe de Jesús and street dust from surrounding settlements. The geogenic end-member field is shown according to the representative lithology of the study area (González-Becuar et al., 2017; González-León et al., 2011). Also shown is the isotope data of the 2014 Buenavista del Cobre mine spill (Romo-Morales et al., 2020) and unleaded (Morton-Bermea et al., 2011) and leaded (Sañudo-Wilhelmy & Flegal, 1994) Mexican gasoline

The undetermination or exclusion of ^204^Pb in environmental studies is common (Komárek et al., 2008) and generally leads to simplistic isotope plots (Chong-López et al., 2024) that may underestimate or overestimate the influence of geogenic or anthropogenic components. To accurately assess the Pb isotope composition of the studied environmental matrices, Fig. 4 includes the isotope data in terms of ^204^Pb. Similarly, the tendency line formed by the mine tailings samples is composed by a less radiogenic end-member represented by the sulfide-rich materials (^206/204^Pb = 18.859–19.113; ^207/204^Pb = 15.639–15.672; ^208/204^Pb = 38.693–38.761) whereas the more radiogenic end-member is represented by efflorescence salts and oxide-rich materials (^206^Pb/^204^Pb = 18.882–19.287; ^207^Pb/^204^Pb = 15.642–15.690; ^208^Pb/^204^Pb = 38.699–38.792), which is located over the geogenic field (Fig. 4).

The Pb isotope trend formed by the less radiogenic ratios toward the more radiogenic values can be explained by the oxidation of sulfide minerals that triggered the formation of AMD. The acidification of the tailings materials promoted the release of Pb from sulfides but also from the lithogenic components included in the tailings, such as sediments, minerals, and rock fragments (e.g., altered rocks that are highly susceptible to leaching by AMD). The geogenic component is characterized by higher radiogenic Pb ratios (i.e., geogenic end-member) and is associated with the silicate minerals (i.e., rock forming minerals). Therefore, the linear trend observed in the mine talings materials is the result of mixing between Pb from sulfide-rich materials and lithogenic Pb (Fig. 4).

An important finding is that the Pb isotope composition of the street dust from San Felipe de Jesús town is intimately associated with the tendency line formed by the isotope compositions of the tailings materials, which support wind dispersion of fine-grained materials from the tailings deposit as previously suggested by the presence of rozenite (FeSO_4_ ⋅ 4H_2_O), a secondary hydrous iron sulfate mineral identified in the tailings deposit and the street dust from San Felipe de Jesús settlement (Del Rio-Salas et al., 2019). This evidence supports the dispersion and fate of contaminants related to mine tailings deposits, particularly in arid and semiarid regions, where climate conditions significantly influence the dispersion of materials (Mokhtari et al., 2018; Navarro et al., 2008; Punia, 2021). Excepting one sample from Huépac, the isotope compositions of the street dust samples from Ranchito de Huépac and Huépac settlements, located 5 and 8 km, respectively, northeast of the tailings deposit (Fig. 1), are included along the tendency line formed by the mine tailings materials (Figs. 3 and 4); the isotope composition supports dispersion along a northeast trend, which is the predominant wind direction (Fig. 1). In contrast, the isotope compositions of two street dust samples from Aconchi and one street dust sample from Huépac are not aligned with the tendency line (Fig. 4), indicating the influence of additional Pb sources, for instance, from rural and municipal waste, pesticides and herbicides used in agricultural activity, leaded/unleaded gasoline (e.g., Chrastný et al., 2018; Civitillo et al., 2016; Eichler et al., 2015; Escobar et al., 2013). The isotope composition of these samples exhibits a subtle inclination toward the Pb isotope compositions of Mexican gasoline (Morton-Bremea et al., 2011; Sañudo-Wilhemly and Flegal, 1994), implying a probable influence.

Among the relevant economic activities along the Sonoran River Basin, mining can contribute pollutants to the river plain. The potential contribution can be exacerbated in arid and semiarid regions, particularly during the dry seasons. If river sediments are impacted, suspension of PTE-bearing fine-grained materials can transport pollutants by wind (e.g., Moreno-Rodríguez et al., 2015), or impacted sediments can migrate downstream. Considering the upstream spill of the Buenavista del Cobre mine in 2014, Fig. 4 shows that the Pb isotope compositions of the mine spill and impacted sediments are notably different from the studied street dust, indicating the unlikely influence of such spill over the rural dust. Regarding the Pb isotope composition of agricultural soil samples, they are included in the geogenic isotope field (Fig. 4), suggesting the close influence of the local lithology and the region’s soils.

### Pb traceability from mine tailings deposits

One of the findings of this research highlights the importance of efflorescent salts in terms of metal traceability. Notably, the Pb isotope composition effectively constrains the signature of sulfide-rich tailings and their respective efflorescent salts. In addition, the Pb isotope composition of the efflorescent salts indicates their sensitivity to oxidation and the duration of exposure to weathering. As a result, the longer the tailings have been subjected to weathering, the more oxidized they become, leading to a Pb isotope composition similar to that of the geogenic member, as mixing with geogenic Pb is more likely under such conditions. Therefore, if efflorescent salts are formed from oxidized tailings, Pb involved in the formation of such salts will consist of a Pb mix from sulfide-rich tailings with minerals and rocks from tailings deposits, yielding isotope composition closer to the geogenic end-member (i.e., more radiogenic). In contrast, efflorescent salts formed over the sulfide-rich or slightly oxidized tailings will produce a less radiogenic composition.

Pb traceability of efflorescent salts and oxidized mine tailings might be challenging since tailings materials are heterogeneous and geochemically complex matrices. As a consequence of the oxidation processes in arid and semi-arid environments, mine tailings can yield similar isotope composition than the geogenic end-member, which masks compositions and potentially can underestimate the influence of tailings materials over surrounding media (e.g., soils, dust, sediments). Combining mineralogical evidence, metal content, and Pb isotope composition of efflorescent salts will be crucial in accurately identifying the influence of mine tailings on environmental matrices and human health.

## Conclusions

By using the Pb isotope systematics, it is possible to identify the anthropogenic component (less radiogenic), represented by the sulfide-rich materials and respective efflorescent salts. In contrast, the Pb isotope composition of the more oxidized tailings and respective efflorescent salts is more radiogenic, trending through, and similar to the geogenic end-member. The isotope composition of street dust of the nearby settlements suggests the dispersion of the tailings materials to the surroundings. The variability of the Pb isotope composition (from less through more radiogenic) found in the efflorescent salts might be challenging when tracing pollutants in arid and semi-arid environments, especially when the geogenic member conceals the composition.

The efflorescence salts in mine tailings from either historical or current mining highlight the importance of assessing the geochemical behavior to establish stabilizing procedures to avoid PTE dispersal to environmental media, considering the hazard represented by the presence of PTE hyperaccumulators and highly soluble efflorescent salts. Therefore, tracking the source, dispersion, and fate of pollutants during environmental assessments of mine-related waste from arid- and semi-arid environments is crucial. Equally important are the government’s actions in establishing guidelines (e.g., characterization, mitigation, remediation, regulations) to ensure that efflorescent salts do not pose environmental and health risks.

## Supplementary Information

Below is the link to the electronic supplementary material.

Supplementary file1 (DOCX 43 KB)

## Data Availability

No datasets were generated or analysed during the current study.
