# Supplementary material for: Traceability and dispersion of highly toxic soluble phases from historical mine tailings: insights from Pb isotope systematics
Source: Environ Geochem Health. 2024 Aug 24;46(10):395. doi: 10.1007/s10653-024-02180-3 (PMC11344714; doi:10.1007/s10653-024-02180-3)
Supplement: Supplementary file 1 — Supplementary file1 (DOCX 43 KB) [file 10653_2024_2180_MOESM1_ESM.docx]

**Traceability and dispersion of highly toxic soluble phases from historical mine tailings**

Rafael Del Rio-Salas^1,2^, Verónica Moreno-Rodríguez^3^, René Loredo-Portales^1,2^, Sergio Adrián Salgado-Souto^4^, Martín Valencia-Moreno^1^, Lucas Ochoa-Landín^5^, Diana Romo-Morales^5^

^1^ Estación Regional del Noroeste, Instituto de Geología, Universidad Nacional Autónoma de México, Colosio y Madrid s/n, Hermosillo, Sonora, México, C.P. 83000.

^2^ Laboratorio Nacional de Geoquímica y Mineralogía-LANGEM, Ciudad de México, México, C.P. 04510.

^3^ Ingeniería en Geociencias, Universidad Estatal de Sonora, Av. Ley Federal del Trabajo s/n, Col. Apolo, Hermosillo, Sonora, México, C.P. 83100‬.‬‬‬‬‬‬‬‬‬‬‬‬

^4^ Escuela Superior de Ciencias de la Tierra, Universidad Autónoma de Guerrero, Ex-hacienda de San Juan Bautista s/n, Taxco el Viejo, Guerrero, México, C.P. 40323.

^5^ Departamento de Geología, División de Ciencias Exactas y Naturales, Universidad de Sonora, Rosales y Encinas s/n, Hermosillo, Sonora, México, C.P. 83000.

* Corresponding author: rdelriosalas@gmail.com

Lead isotope raw data of mine tailings and dust samples from San Fleipe de jesús mine site and surrounding areas.

| **Sample** | **208Pb/206Pb (RAW)** | **207Pb/206Pb (RAW)** | **208Pb/207Pb (RAW)** | **206Pb/204Pb (RAW)** | **207Pb/204Pb (RAW)** | **208Pb/204Pb (RAW)** |
| --- | --- | --- | --- | --- | --- | --- |
| JNO3 | 2.0707 | 0.8318 | 2.4896 | 19.1428 | 15.9224 | 39.6398 |
| JNO4 | 2.0748 | 0.8333 | 2.4897 | 19.1033 | 15.9198 | 39.6355 |
| JNO15 | 2.0740 | 0.8330 | 2.4898 | 19.1139 | 15.9222 | 39.6422 |
| JNO8 | 2.0717 | 0.8321 | 2.4896 | 19.1309 | 15.9198 | 39.6331 |
| JG16-02 | 2.0759 | 0.8341 | 2.4888 | 19.0733 | 15.9085 | 39.5930 |
| JSF15-08 | 2.0531 | 0.8248 | 2.4892 | 19.3425 | 15.9538 | 39.7134 |
| JNO10 | 2.0772 | 0.8343 | 2.4898 | 19.0803 | 15.9176 | 39.6324 |
| JSF15-09 | 2.0381 | 0.8191 | 2.4882 | 19.4908 | 15.9653 | 39.7253 |
| HO5 | 2.0356 | 0.8182 | 2.4878 | 19.5167 | 15.9687 | 39.7284 |
| JO1 | 2.0388 | 0.8194 | 2.4882 | 19.4750 | 15.9576 | 39.7048 |
| JO2 | 2.0746 | 0.8333 | 2.4896 | 19.1023 | 15.9178 | 39.6303 |
| JO4 | 2.0506 | 0.8237 | 2.4896 | 19.3589 | 15.9461 | 39.6995 |
| JR16-01 | 2.0733 | 0.8331 | 2.4886 | 19.0954 | 15.9080 | 39.5896 |
| JR16-02 | 2.0675 | 0.8309 | 2.4885 | 19.1516 | 15.9119 | 39.5965 |
| JM16-03 | 2.0682 | 0.8311 | 2.4886 | 19.1526 | 15.9168 | 39.6104 |
| JSF15-03 | 2.0664 | 0.8304 | 2.4885 | 19.1619 | 15.9126 | 39.5963 |
| JSF15-10 | 2.0711 | 0.8319 | 2.4896 | 19.1444 | 15.9260 | 39.6495 |
| EF16-01 | 2.0695 | 0.8316 | 2.4885 | 19.1269 | 15.9064 | 39.5838 |
| JSF15-14 | 2.0475 | 0.8231 | 2.4875 | 19.3647 | 15.9392 | 39.6491 |
| ENO1 | 2.0692 | 0.8312 | 2.4894 | 19.1589 | 15.9245 | 39.6440 |
| EO2 | 2.0382 | 0.8191 | 2.4882 | 19.4916 | 15.9659 | 39.7270 |
| EO1 | 2.0356 | 0.8182 | 2.4879 | 19.5113 | 15.9641 | 39.7180 |
| JSF15-10 | 2.0676 | 0.8309 | 2.4884 | 19.1575 | 15.9178 | 39.6105 |
| JSF15-12B | 2.0368 | 0.8186 | 2.4882 | 19.5026 | 15.9642 | 39.7216 |
| ACN-01 | 2.0654 | 0.8327 | 2.4803 | 19.1273 | 15.9275 | 39.5052 |
| ACN-02 | 2.0578 | 0.8276 | 2.4863 | 19.2455 | 15.9282 | 39.6020 |
| ACN-03 | 2.0644 | 0.8317 | 2.4822 | 19.1508 | 15.9266 | 39.5353 |
| HPC-01 | 2.0495 | 0.8243 | 2.4862 | 19.3386 | 15.9417 | 39.6344 |
| HPC-02a | 2.0706 | 0.8322 | 2.4880 | 19.1177 | 15.9107 | 39.5852 |
| HPC-02b | 2.0705 | 0.8322 | 2.4880 | 19.1172 | 15.9092 | 39.5823 |
| HPC-03 | 2.0509 | 0.8258 | 2.4835 | 19.2989 | 15.9362 | 39.5783 |
| RHPC-01 | 2.0480 | 0.8229 | 2.4888 | 19.3708 | 15.9400 | 39.6715 |
| PSF16-01 | 2.0550 | 0.8264 | 2.4867 | 19.2752 | 15.9292 | 39.6109 |
| PSF16-02 | 2.0497 | 0.8241 | 2.4871 | 19.3315 | 15.9322 | 39.6231 |
| PSF16-03 | 2.0555 | 0.8265 | 2.4870 | 19.2630 | 15.9204 | 39.5951 |
| PSF16-04 | 2.0690 | 0.8316 | 2.4881 | 19.1358 | 15.9125 | 39.5924 |
| **Sample** | **208Pb/206Pb (RAW)** | **207Pb/206Pb (RAW)** | **208Pb/207Pb (RAW)** | **206Pb/204Pb (RAW)** | **207Pb/204Pb (RAW)** | **208Pb/204Pb (RAW)** |
| PSF16-06 | 2.0377 | 0.8194 | 2.4868 | 19.4658 | 15.9506 | 39.6655 |
| PSF16-07 | 2.0395 | 0.8200 | 2.4870 | 19.4399 | 15.9418 | 39.6463 |
| PSF16-08 | 2.0515 | 0.8247 | 2.4875 | 19.3188 | 15.9324 | 39.6324 |
| SRSF-01 | 2.0689 | 0.8302 | 2.4922 | 19.1583 | 15.9047 | 39.6383 |
| SRSF-02 | 2.1087 | 0.8485 | 2.4853 | 18.6880 | 15.8559 | 39.4070 |

Replicates of lead isotope ratios of NIST (NBS) 981.

| **208Pb/206Pb** | **207Pb/206Pb** | **208Pb/207Pb** | **206Pb/204Pb** | **207Pb/204Pb** | **208Pb/204Pb** |
| --- | --- | --- | --- | --- | --- |
| 2.167803257 | 0.914796483 | 2.36970311 | 16.9408409 | 15.4974917 | 36.7238087 |
| 2.167688306 | 0.914777643 | 2.36964877 | 16.9397038 | 15.4955068 | 36.7196553 |
| 2.167770997 | 0.914807704 | 2.36966159 | 16.9405548 | 15.4972603 | 36.7228197 |
| 2.167504824 | 0.914736521 | 2.36958168 | 16.9384373 | 15.4940282 | 36.7137991 |
| 2.167717293 | 0.914786278 | 2.36962973 | 16.9415479 | 15.498123 | 36.7255759 |
| 2.167670071 | 0.914755156 | 2.36970105 | 16.93992 | 15.4961285 | 36.7197048 |
| 2.167862094 | 0.91478348 | 2.36977026 | 16.9449059 | 15.50037 | 36.7338001 |
| 2.1676649 | 0.914764524 | 2.36961476 | 16.9393036 | 15.4956194 | 36.7180779 |
| 2.167801109 | 0.914782783 | 2.36970524 | 16.9395844 | 15.4960903 | 36.7222876 |
| 2.167754558 | 0.914759066 | 2.36979026 | 16.9393821 | 15.4951411 | 36.7203506 |
| 2.16783662 | 0.914798656 | 2.3696918 | 16.9425851 | 15.4991311 | 36.7275829 |
| 2.167611813 | 0.914750329 | 2.36960806 | 16.9403914 | 15.4963166 | 36.7201187 |
| 2.167932482 | 0.9148272 | 2.36978974 | 16.9422181 | 15.4988117 | 36.7285806 |
| 2.16764213 | 0.9147499 | 2.36966109 | 16.9403811 | 15.4969503 | 36.7211445 |
| 2.167583909 | 0.914715412 | 2.36965687 | 16.9386863 | 15.4942732 | 36.7157476 |
| 2.167707235 | 0.914780716 | 2.36966965 | 16.9413072 | 15.4976968 | 36.7245622 |
| 2.167662298 | 0.914745994 | 2.36967681 | 16.9389321 | 15.4944452 | 36.7176816 |
| 2.167815881 | 0.914735203 | 2.36986287 | 16.9441449 | 15.4996268 | 36.7314141 |
| 2.167262019 | 0.914627073 | 2.36957798 | 16.9400556 | 15.4935552 | 36.7128379 |
| 2.167647436 | 0.914682379 | 2.36976942 | 16.9416911 | 15.4963519 | 36.7230538 |
| 2.167616738 | 0.914691263 | 2.36975156 | 16.9427136 | 15.4975411 | 36.7249575 |
| 2.167715634 | 0.91473264 | 2.36974847 | 16.9419351 | 15.497258 | 36.725654 |
| 2.167763823 | 0.91475827 | 2.36979358 | 16.940577 | 15.4965882 | 36.7236044 |
| 2.167749824 | 0.914731312 | 2.36980969 | 16.9420342 | 15.4974487 | 36.7258161 |
| 2.167689522 | 0.914735762 | 2.3697328 | 16.9385826 | 15.4946918 | 36.7168345 |
| 2.167647349 | 0.914737056 | 2.36969117 | 16.9377923 | 15.4936867 | 36.714877 |
| 2.167501344 | 0.914710688 | 2.36958969 | 16.9397571 | 15.495106 | 36.7175661 |
| 2.167916876 | 0.91479244 | 2.36985641 | 16.942554 | 15.4994256 | 36.7305484 |
| 2.167777568 | 0.914762167 | 2.36971997 | 16.9398484 | 15.4959245 | 36.7216524 |
| 2.167821957 | 0.91478603 | 2.36976318 | 16.9442029 | 15.4998112 | 36.7310485 |
| 2.167692682 | 0.914763269 | 2.3696894 | 16.9392962 | 15.4951881 | 36.7180825 |
| **208Pb/206Pb** | **207Pb/206Pb** | **208Pb/207Pb** | **206Pb/204Pb** | **207Pb/204Pb** | **208Pb/204Pb** |
| 2.167857929 | 0.914777645 | 2.36980894 | 16.9390113 | 15.4957653 | 36.7222892 |
| 2.167742371 | 0.914730152 | 2.36983298 | 16.9408666 | 15.4963009 | 36.7228593 |
| 2.167670936 | 0.914735513 | 2.36975704 | 16.9389287 | 15.4945749 | 36.7180427 |
| 2.167693711 | 0.914746638 | 2.36975517 | 16.9408476 | 15.496537 | 36.7224583 |
| 2.167829428 | 0.914781822 | 2.36981986 | 16.9410029 | 15.4970619 | 36.7247056 |
| 2.167511979 | 0.914671776 | 2.36973435 | 16.9394565 | 15.4938929 | 36.7153574 |
| 2.167725735 | 0.9147271 | 2.36983117 | 16.9394115 | 15.4944944 | 36.7192666 |
| 2.167575023 | 0.914701913 | 2.36970782 | 16.9387994 | 15.493615 | 36.7173166 |
| 2.167700909 | 0.914719119 | 2.36981564 | 16.9380663 | 15.4935796 | 36.7162973 |
| 2.167954147 | 0.914797935 | 2.36989628 | 16.9395398 | 15.4961614 | 36.7237551 |
| 2.167593202 | 0.914794302 | 2.36947535 | 16.9414605 | 15.4981416 | 36.7220713 |
| 2.167593202 | 0.914794302 | 2.36947535 | 16.9414605 | 15.4981416 | 36.7220713 |
| 2.167792476 | 0.914801801 | 2.369705 | 16.9390199 | 15.495855 | 36.7212075 |
| 2.167775671 | 0.914798276 | 2.36969696 | 16.9420596 | 15.4984088 | 36.7268924 |
| 2.167617486 | 0.914824658 | 2.369447 | 16.9358239 | 15.4934819 | 36.7112522 |
| 2.167512558 | 0.914770466 | 2.36949011 | 16.9391791 | 15.4949855 | 36.7152706 |
| 2.167543594 | 0.914745147 | 2.36949625 | 16.9379476 | 15.4940818 | 36.7125146 |
| 2.167832831 | 0.914827728 | 2.3696707 | 16.9415254 | 15.4985652 | 36.7256395 |
| 2.167986919 | 0.914838237 | 2.36977926 | 16.9409113 | 15.4976524 | 36.7264904 |
| 2.167760464 | 0.914794314 | 2.36966246 | 16.9412995 | 15.4972348 | 36.7244944 |
| 2.167660382 | 0.914773736 | 2.3696202 | 16.9400214 | 15.4961597 | 36.7185332 |
| 2.167790731 | 0.914807482 | 2.3696522 | 16.9379294 | 15.4952144 | 36.718918 |
| 2.167911858 | 0.914825223 | 2.36974601 | 16.9401549 | 15.4971392 | 36.7248669 |
| 2.167813173 | 0.914771805 | 2.36978724 | 16.9384175 | 15.495479 | 36.7206033 |
| 2.167836539 | 0.914767476 | 2.36984078 | 16.9389507 | 15.4944955 | 36.7208955 |
| 2.167770567 | 0.914762415 | 2.36979666 | 16.9375139 | 15.4938042 | 36.7158414 |
| 2.167881469 | 0.914788055 | 2.36986474 | 16.939091 | 15.4952831 | 36.7209483 |
| 2.167713491 | 0.914765115 | 2.36967731 | 16.9425846 | 15.4986306 | 36.7249893 |
| 2.167557828 | 0.914685303 | 2.3697245 | 16.9436097 | 15.4983806 | 36.7266105 |
| 2.167665586 | 0.914719205 | 2.36978563 | 16.943686 | 15.498903 | 36.728502 |
| 2.167532017 | 0.914690407 | 2.36968243 | 16.9389042 | 15.4937419 | 36.7144822 |
| 2.167673058 | 0.914736095 | 2.36974851 | 16.9418199 | 15.4976595 | 36.7238119 |
| 2.167531287 | 0.914719503 | 2.36962783 | 16.9385556 | 15.4939104 | 36.7171227 |
| 2.167703782 | 0.914743905 | 2.36972988 | 16.940185 | 15.4961103 | 36.7209519 |
| 2.167630914 | 0.914715696 | 2.36973865 | 16.9428309 | 15.4978578 | 36.7256487 |
| 2.167818236 | 0.914748498 | 2.36982672 | 16.9420659 | 15.497995 | 36.7275728 |
| 2.167855382 | 0.914764739 | 2.36986207 | 16.9412774 | 15.4964841 | 36.7245054 |
| 2.167690157 | 0.914722676 | 2.36977965 | 16.9409658 | 15.4959342 | 36.7222153 |
| 2.167634332 | 0.914686534 | 2.36981622 | 16.9421212 | 15.4962639 | 36.7234296 |
| **208Pb/206Pb** | **207Pb/206Pb** | **208Pb/207Pb** | **206Pb/204Pb** | **207Pb/204Pb** | **208Pb/204Pb** |
| 2.167740185 | 0.914720325 | 2.36984724 | 16.9417346 | 15.4972248 | 36.7259943 |
| 2.167431103 | 0.914637024 | 2.36970932 | 16.9395665 | 15.493411 | 36.7153779 |
| 2.167635098 | 0.914721831 | 2.36969434 | 16.9377609 | 15.4933488 | 36.714847 |
| 2.167713798 | 0.914748857 | 2.36974941 | 16.9411909 | 15.4968657 | 36.7236101 |
| 2.167841029 | 0.914762324 | 2.36985983 | 16.9382604 | 15.4951943 | 36.7202225 |
| 2.16771401 | 0.914735895 | 2.36976446 | 16.9418567 | 15.4972463 | 36.7240411 |
| 2.167590627 | 0.914715185 | 2.36972393 | 16.93945 | 15.494867 | 36.7182281 |
| 2.167746327 | 0.914726085 | 2.36982447 | 16.9423663 | 15.4975916 | 36.726304 |
| 2.167686644 | 0.914714351 | 2.36982935 | 16.939898 | 15.4955405 | 36.7208105 |
| 2.16765093 | 0.914708364 | 2.369753 | 16.94022 | 15.494972 | 36.7199679 |
| 2.167844545 | 0.9147392 | 2.36990531 | 16.9422285 | 15.4974688 | 36.7274056 |
| 2.167875721 | 0.914745199 | 2.36987853 | 16.9423397 | 15.4981017 | 36.7282314 |
| 2.167728297 | 0.914692625 | 2.36982621 | 16.9415965 | 15.4962844 | 36.7248562 |
| 2.167788088 | 0.914806045 | 2.36969988 | 16.9416847 | 15.4978161 | 36.7263462 |
| 2.167687888 | 0.914782072 | 2.3696171 | 16.9419434 | 15.498415 | 36.7247145 |
| 2.16788402 | 0.914813628 | 2.36973782 | 16.9399441 | 15.4976773 | 36.7255012 |
| 2.167737764 | 0.914783724 | 2.36965722 | 16.9427411 | 15.4983531 | 36.7260837 |
| 2.16771277 | 0.914771377 | 2.36968488 | 16.9383959 | 15.494536 | 36.7185637 |
| 2.167651397 | 0.914755638 | 2.36964547 | 16.9424527 | 15.4978713 | 36.7248003 |
| 2.167526466 | 0.914758031 | 2.36949289 | 16.9414737 | 15.497237 | 36.719492 |
| 2.167684091 | 0.914776308 | 2.3696302 | 16.9427072 | 15.4987079 | 36.7261996 |
| 2.167598238 | 0.914736841 | 2.36959956 | 16.942343 | 15.4976494 | 36.7226034 |
| 2.167741845 | 0.914779895 | 2.36966196 | 16.9404324 | 15.4968128 | 36.7221606 |
| 2.167684007 | 0.914771101 | 2.36964488 | 16.9393188 | 15.4962167 | 36.719796 |
| 2.167661147 | 0.914754629 | 2.36965978 | 16.9429703 | 15.4994138 | 36.7265532 |
| 2.167512366 | 0.914747482 | 2.36952356 | 16.9401998 | 15.4967473 | 36.7169832 |
| 2.16783616 | 0.914818662 | 2.36966142 | 16.9400077 | 15.4972925 | 36.722958 |
| 2.1676699 | 0.914745708 | 2.36971703 | 16.9423175 | 15.4979603 | 36.726387 |
| 2.167523722 | 0.914708276 | 2.36960922 | 16.9395637 | 15.4949125 | 36.7166113 |
| 2.167919304 | 0.914818435 | 2.36978296 | 16.944243 | 15.5002243 | 36.7316494 |
| 2.167624186 | 0.914735049 | 2.36968825 | 16.9411101 | 15.4969541 | 36.7215905 |
| 2.167770938 | 0.914785366 | 2.36968384 | 16.9407299 | 15.4971438 | 36.7232492 |
| 2.167594954 | 0.914739139 | 2.36962229 | 16.9395797 | 15.495184 | 36.7170825 |
| 2.167871799 | 0.91482393 | 2.36973374 | 16.9424405 | 15.4988757 | 36.7280397 |
| 2.167670421 | 0.914782938 | 2.36963174 | 16.9402089 | 15.4962739 | 36.7205404 |
| 2.167933466 | 0.914815621 | 2.36982037 | 16.9408961 | 15.4978729 | 36.7264665 |
| 2.167738368 | 0.91476882 | 2.36966739 | 16.9382221 | 15.4943992 | 36.7167056 |
| 2.167759266 | 0.914800013 | 2.36963192 | 16.9407317 | 15.4974948 | 36.7237755 |
| 2.167679564 | 0.914776615 | 2.3696527 | 16.9395754 | 15.4959028 | 36.7200276 |
| **208Pb/206Pb** | **207Pb/206Pb** | **208Pb/207Pb** | **206Pb/204Pb** | **207Pb/204Pb** | **208Pb/204Pb** |
| 2.167600389 | 0.914720718 | 2.36967517 | 16.9390845 | 15.4949609 | 36.7172037 |
| 2.167699164 | 0.914745773 | 2.36972175 | 16.9425109 | 15.4979009 | 36.7258668 |
| 2.167686535 | 0.914738251 | 2.36975081 | 16.9355431 | 15.4918012 | 36.7117517 |
| 2.167650571 | 0.914727207 | 2.36973239 | 16.9416747 | 15.4967294 | 36.7228533 |
| 2.167699327 | 0.914756703 | 2.36970253 | 16.9381414 | 15.4943096 | 36.7167956 |
| 2.167655244 | 0.914772389 | 2.36966816 | 16.9403017 | 15.4962922 | 36.720726 |
| 2.167852419 | 0.914784092 | 2.3698396 | 16.9400569 | 15.4961016 | 36.723401 |
| 2.167611646 | 0.914715893 | 2.3697213 | 16.9388798 | 15.4933079 | 36.7155864 |
| 2.167474651 | 0.914663874 | 2.3697003 | 16.9379495 | 15.4924373 | 36.7118861 |
| 2.16746759 | 0.914676935 | 2.36965969 | 16.9377623 | 15.4924265 | 36.710765 |
| 2.167584358 | 0.914686214 | 2.36978704 | 16.9403632 | 15.4946227 | 36.7197183 |
| 2.167501897 | 0.914641988 | 2.36976043 | 16.9403602 | 15.494904 | 36.7201147 |
| 2.16773984 | 0.914744496 | 2.36979305 | 16.9390024 | 15.4940419 | 36.7188083 |
| 2.167601536 | 0.914693118 | 2.36974765 | 16.9386568 | 15.4934104 | 36.7157526 |
| 2.16778529 | 0.914696108 | 2.36993815 | 16.9399561 | 15.4951603 | 36.7220908 |
| 2.167744739 | 0.914707016 | 2.36988308 | 16.9429607 | 15.4975915 | 36.7281077 |
| 2.167672538 | 0.91468852 | 2.36986804 | 16.9391643 | 15.495316 | 36.7209335 |
| 2.167750201 | 0.914685053 | 2.36993396 | 16.9404337 | 15.4954426 | 36.7221172 |
| 2.167744472 | 0.914684087 | 2.36994049 | 16.9402483 | 15.4949128 | 36.7215243 |
| 2.167967798 | 0.914768269 | 2.36999091 | 16.9413133 | 15.4969291 | 36.7282773 |
| 2.167907093 | 0.914743504 | 2.36993192 | 16.9409679 | 15.4965373 | 36.7263287 |
| 2.167910065 | 0.914760265 | 2.36991514 | 16.9412398 | 15.4978348 | 36.728907 |
| 2.167905357 | 0.914765814 | 2.3699265 | 16.9426307 | 15.4983528 | 36.7299802 |
| 2.167788088 | 0.914806045 | 2.36969988 | 16.9416847 | 15.4978161 | 36.7263462 |
| 2.167687888 | 0.914782072 | 2.3696171 | 16.9419434 | 15.498415 | 36.7247145 |
| 2.16788402 | 0.914813628 | 2.36973782 | 16.9399441 | 15.4976773 | 36.7255012 |
| 2.167737764 | 0.914783724 | 2.36965722 | 16.9427411 | 15.4983531 | 36.7260837 |
| 2.16771277 | 0.914771377 | 2.36968488 | 16.9383959 | 15.494536 | 36.7185637 |
| 2.167651397 | 0.914755638 | 2.36964547 | 16.9424527 | 15.4978713 | 36.7248003 |
| 2.167526466 | 0.914758031 | 2.36949289 | 16.9414737 | 15.497237 | 36.719492 |
| 2.167684091 | 0.914776308 | 2.3696302 | 16.9427072 | 15.4987079 | 36.7261996 |
| 2.167598238 | 0.914736841 | 2.36959956 | 16.942343 | 15.4976494 | 36.7226034 |
| 2.167741845 | 0.914779895 | 2.36966196 | 16.9404324 | 15.4968128 | 36.7221606 |
| 2.167684007 | 0.914771101 | 2.36964488 | 16.9393188 | 15.4962167 | 36.719796 |
| 2.167661147 | 0.914754629 | 2.36965978 | 16.9429703 | 15.4994138 | 36.7265532 |
| 2.167512366 | 0.914747482 | 2.36952356 | 16.9401998 | 15.4967473 | 36.7169832 |
| 2.16783616 | 0.914818662 | 2.36966142 | 16.9400077 | 15.4972925 | 36.722958 |
| 2.1676699 | 0.914745708 | 2.36971703 | 16.9423175 | 15.4979603 | 36.726387 |
| 2.167523722 | 0.914708276 | 2.36960922 | 16.9395637 | 15.4949125 | 36.7166113 |
| **208Pb/206Pb** | **207Pb/206Pb** | **208Pb/207Pb** | **206Pb/204Pb** | **207Pb/204Pb** | **208Pb/204Pb** |
| 2.167624186 | 0.914735049 | 2.36968825 | 16.9411101 | 15.4969541 | 36.7215905 |
| 2.167770938 | 0.914785366 | 2.36968384 | 16.9407299 | 15.4971438 | 36.7232492 |
| 2.167594954 | 0.914739139 | 2.36962229 | 16.9395797 | 15.495184 | 36.7170825 |
| 2.167871799 | 0.91482393 | 2.36973374 | 16.9424405 | 15.4988757 | 36.7280397 |
| 2.167670421 | 0.914782938 | 2.36963174 | 16.9402089 | 15.4962739 | 36.7205404 |
| 2.167933466 | 0.914815621 | 2.36982037 | 16.9408961 | 15.4978729 | 36.7264665 |
| 2.167738368 | 0.91476882 | 2.36966739 | 16.9382221 | 15.4943992 | 36.7167056 |
| 2.167759266 | 0.914800013 | 2.36963192 | 16.9407317 | 15.4974948 | 36.7237755 |
| 2.167679564 | 0.914776615 | 2.3696527 | 16.9395754 | 15.4959028 | 36.7200276 |
| 2.167825148 | 0.914778369 | 2.36972559 | 16.9395645 | 15.4957318 | 36.7206232 |
| 2.167600389 | 0.914720718 | 2.36967517 | 16.9390845 | 15.4949609 | 36.7172037 |
| 2.167699164 | 0.914745773 | 2.36972175 | 16.9425109 | 15.4979009 | 36.7258668 |
| 2.167686535 | 0.914738251 | 2.36975081 | 16.9355431 | 15.4918012 | 36.7117517 |
| 2.167650571 | 0.914727207 | 2.36973239 | 16.9416747 | 15.4967294 | 36.7228533 |
| 2.167699327 | 0.914756703 | 2.36970253 | 16.9381414 | 15.4943096 | 36.7167956 |
| 2.167655244 | 0.914772389 | 2.36966816 | 16.9403017 | 15.4962922 | 36.720726 |
| 2.167852419 | 0.914784092 | 2.3698396 | 16.9400569 | 15.4961016 | 36.723401 |
| 2.167611646 | 0.914715893 | 2.3697213 | 16.9388798 | 15.4933079 | 36.7155864 |
| 2.167474651 | 0.914663874 | 2.3697003 | 16.9379495 | 15.4924373 | 36.7118861 |
| 2.16746759 | 0.914676935 | 2.36965969 | 16.9377623 | 15.4924265 | 36.710765 |
| 2.167584358 | 0.914686214 | 2.36978704 | 16.9403632 | 15.4946227 | 36.7197183 |
| 2.167501897 | 0.914641988 | 2.36976043 | 16.9403602 | 15.494904 | 36.7201147 |
| 2.16773984 | 0.914744496 | 2.36979305 | 16.9390024 | 15.4940419 | 36.7188083 |
| 2.167601536 | 0.914693118 | 2.36974765 | 16.9386568 | 15.4934104 | 36.7157526 |
| 2.16778529 | 0.914696108 | 2.36993815 | 16.9399561 | 15.4951603 | 36.7220908 |
| 2.167744739 | 0.914707016 | 2.36988308 | 16.9429607 | 15.4975915 | 36.7281077 |
| 2.167672538 | 0.91468852 | 2.36986804 | 16.9391643 | 15.495316 | 36.7209335 |
| 2.167750201 | 0.914685053 | 2.36993396 | 16.9404337 | 15.4954426 | 36.7221172 |
| 2.167744472 | 0.914684087 | 2.36994049 | 16.9402483 | 15.4949128 | 36.7215243 |
| 2.167967798 | 0.914768269 | 2.36999091 | 16.9413133 | 15.4969291 | 36.7282773 |
| 2.167907093 | 0.914743504 | 2.36993192 | 16.9409679 | 15.4965373 | 36.7263287 |
| 2.167910065 | 0.914760265 | 2.36991514 | 16.9412398 | 15.4978348 | 36.728907 |
| 2.167905357 | 0.914765814 | 2.3699265 | 16.9426307 | 15.4983528 | 36.7299802 |
